# Targeting GATA4 for cardiac repair

**DOI:** 10.1002/iub.2150

**Published:** 2019-08-16

**Authors:** Mika J. Välimäki, Heikki J. Ruskoaho

**Affiliations:** ^1^ Drug Research Program, Division of Pharmacology and Pharmacotherapy, Faculty of Pharmacy University of Helsinki Helsinki, Finland

**Keywords:** medicinal chemistry, protein function, transcription factors, transcriptional regulation

## Abstract

Various strategies have been applied to replace the loss of cardiomyocytes in order to restore reduced cardiac function and prevent the progression of heart disease. Intensive research efforts in the field of cellular reprogramming and cell transplantation may eventually lead to efficient in vivo applications for the treatment of cardiac injuries, representing a novel treatment strategy for regenerative medicine. Modulation of cardiac transcription factor (TF) networks by chemical entities represents another viable option for therapeutic interventions. Comprehensive screening projects have revealed a number of molecular entities acting on molecular pathways highly critical for cellular lineage commitment and differentiation, including compounds targeting Wnt‐ and transforming growth factor beta (TGFβ)‐signaling. Furthermore, previous studies have demonstrated that GATA4 and NKX2‐5 are essential TFs in gene regulation of cardiac development and hypertrophy. For example, both of these TFs are required to fully activate mechanical stretch‐responsive genes such as atrial natriuretic peptide and brain natriuretic peptide (BNP). We have previously reported that the compound 3i‐1000 efficiently inhibited the synergy of the GATA4–NKX2‐5 interaction. Cellular effects of 3i‐1000 have been further characterized in a number of confirmatory in vitro bioassays, including rat cardiac myocytes and animal models of ischemic injury and angiotensin II‐induced pressure overload, suggesting the potential for small molecule‐induced cardioprotection.

**Abbreviations**
ANP: atrial natriuretic peptide
BNP: brain natriuretic peptide
ChIP‐seq: chromatin immunoprecipitation‐sequencing
CREP: cAMP‐response element binding protein
ERK: extracellular signal‐regulated kinase
ET‐1: endothelin‐1
GSK‐3β: glycogen synthase kinase 3β
hiPSC: human‐induced pluripotent stem cells
MAPK: mitogen‐activated protein kinase
NMR: nuclear magnetic resonance
PDB: protein data bank
PE: phenylephrine
STAT‐1: signal transducer and activator of transcription‐1
SUMO‐1: small ubiquitin‐like modifier‐1
TF: transcription factor
TGF: transforming growth factor

## 1 INTRODUCTION

A network of cardiac transcription factor (TF) controls cardiac gene expression and has a central role in transcriptional regulation during cardiac differentiation and development and the adaptive pathophysiological processes in the adult heart.^1^, ^2^, ^3^ Evolutionarily conserved cardiac TFs GATA binding protein 4 (GATA4), NK2 homeobox 5 (NKX2‐5), myocyte enhancer factor 2C (MEF2C), heart and neural crest derivatives expressed 2 (HAND2), serum response factor (SRF), and T‐box 5 (TBX5) have been shown to interact with and orchestrate cardiac gene expression during differentiation and development and are also involved in cardiac hypertrophy in a context‐dependent and dynamically evolving manner (Table 1). Increasing evidence shows that a restricted number of regulatory TFs (e.g., GATA4, HAND2, MEF2, NKX2‐5, and TBX5) are required for the initiation of cardiac‐like gene expression and are capable of cooperatively reprogramming cardiac fibroblasts into functional cardiac‐like myocytes in vitro and in vivo.^4^, ^5^, ^6^, ^7^ In particular, the pioneer TF GATA4 has emerged as the nuclear effector of several cardiac signaling pathways that modulate key cardiac cascades through post‐translational modifications and protein–protein interactions.

**Table 1:** Summary of the evolutionarily conserved transcription factors expressed in the heart

Transcription factor	Size	Isoforms	Structural classification	Interacting partners
GATA4	442 aa	2	Zinc finger protein	NKX2‐5, TBX5, MEF2C
NKX2‐5	324 aa	3	Homeobox protein	GATA4, TBX5
MEF2C	473 aa	6	MADS‐box superfamily	GATA4, p300
TBX5	518 aa	3	T‐box protein	GATA4, NKX2‐5
SRF	508 aa	1	MADS‐box superfamily	GATA4, ELK4, myogenin
HAND2	217 aa	2	Basic helix–loop–helix protein	GATA4, NKX2‐5
FOG2	1,151 aa	3	Zinc finger protein	GATA4

In humans, the evolutionarily conserved GATA‐family of proteins consists of six GATA proteins (GATA1‐6), all sharing similar tertiary protein structures and high amino acid sequence identity over their two DNA binding zinc finger domains. Both N‐terminal‐ and C‐terminal zinc atoms in the GATA family are tetrahedrally coordinated and bound to four cysteine residues (Cys_4_) forming the protein domains involving two β‐sheets and one α‐helix. The stable N‐terminal zinc finger of GATA4 is primarily responsible for mediating physical interaction and gene repression via binding to friend of GATA2 protein (FOG2),^8^ whereas the vast majority of the synergistic heterotypic interactions of GATA4 are physically mediated by the C‐terminal zinc finger and its C‐terminal extension.^2^ In comparison to human and animal genomes, the GATA TF families are comparatively large in plant model organisms, with approximately 30 members in *Arabidopsis thaliana* and 64 members in soya beans. Furthermore, a recent study in plants suggests that two important processes during plant development, greening and photosynthesis, as well as stomata formation, and thus, gas exchange, are regulated by plant GATA‐factors.^9^, ^10^

## 2 GATA4 STRUCTURE AND FUNCTION

**Figure 1:**
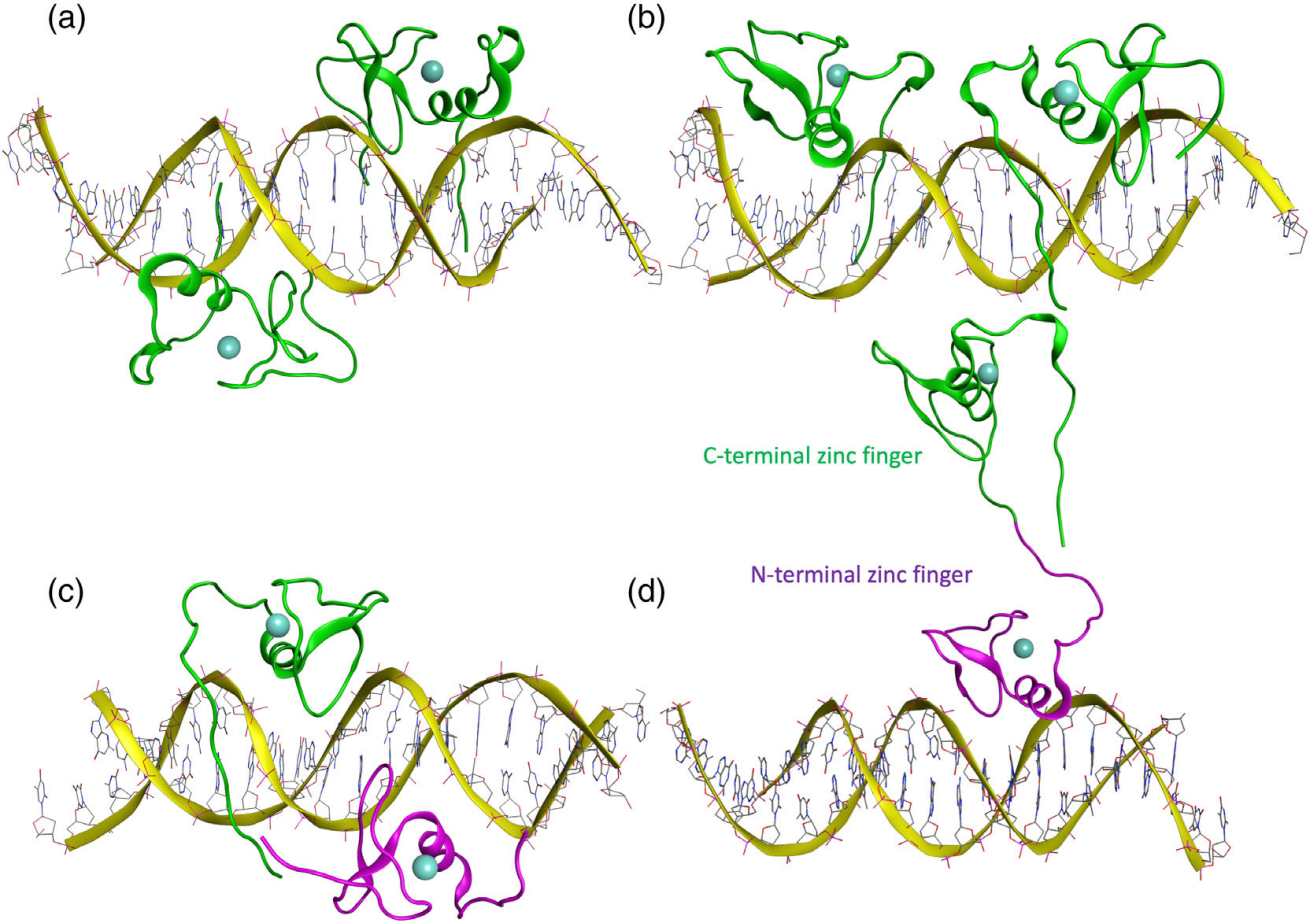
Variable DNA‐binding modes of GATA‐proteins. (a) Opposite DNA‐binding of C‐terminal zinc fingers of GATA3 (PDB, 3DFX), (b) adjacent DNA‐binding of C‐terminal zinc fingers of GATA3 (PDB, 3DFV), (c) both N‐ and C‐terminal zinc fingers of GATA1 bound to palindromic DNA recognition site (PDB, 3VD6), (d) both N‐ and C‐terminal zinc fingers of GATA3 bind on different DNA molecules, thereby bridging two independent and separate DNA fragments suggesting a mechanisms of DNA looping and long‐range gene regulation. This finding was confirmed in solution by an in‐gel fluorescence resonance energy transfer analysis (PDB, 4HC7)^14^

The protein sequence of human GATA4 contains multiple functional domains, including the C‐ and N‐terminal zinc fingers, in addition to the N‐terminal and C‐terminal sequences, which have been suggested to constitute transcriptional activation and nuclear localization domains, respectively.^11^, ^12^ The truncated protein structure of the C‐terminal zinc finger of GATA4 has been experimentally resolved with nuclear magnetic resonance (NMR) by the Northeast Structural Genomics Consortium (protein data bank [PDB] code, 2M9W). However, the first NMR‐structures of the zinc finger domain for GATA1 (PDB, 1GAT) were published in 1993.^13^ Furthermore, X‐ray crystallographic binding analyses of other GATA‐family zinc fingers bound to DNA have provided new insights into the DNA recognition mechanisms of GATA‐dependent gene regulation (Figure 1).^14^, ^15^

The human GATA4 protein contains 442 amino acids and includes two structurally stable zinc finger domains located at amino acid residues 217–241 and 271–295. The protein sequence outside of the zinc finger core domains and C‐terminal extension (residues 210–320) has no globular structure and it remains completely disordered.^16^ In addition, the C‐terminal extension of the zinc finger contains the amino acid sequence required for nuclear localization.^12^ Several GATA4 single point mutations identified from humans have been shown to be linked to common developmental anomalies and mortality in new‐borns. For example, the heterozygous G296S missense mutation of GATA4 results in diminished DNA binding affinity, diminished transcriptional activity, and abolition of a physical interaction between GATA4 and TBX5 that is associated with congenital heart disease.^17^, ^18^ Moreover, four heterozygous missense GATA4 mutations, P36S, H190R, S262A, and V399G, have been linked to congenital atrial‐septal defects in new‐borns and are responsible for substantial morbidity and mortality in affected individuals.^19^

The GATA4 protein was originally discovered as one of the earliest molecular markers associated with the initiation of cardiac gene expression.^20^, ^21^, ^22^ In addition to the heart, GATA4/5/6 proteins are expressed in various tissues, including the liver, lung, gut, and gonad.^12^, ^23^, ^24^ The other GATA family proteins, GATA1/2/3, are preferentially expressed in hematopoietic cells.^25^ Knockout studies of GATA4/5/6 proteins during embryonic heart development in Xenopus, zebrafish, and mice propose a functional redundancy between these TFs. Moreover, a number of studies have demonstrated that none of the GATA factors are absolutely required for the specification of myocardium, suggesting a compensatory mechanism inside the GATA family.^26^, ^27^, ^28^

On one hand, it appears that total control of GATA4 mRNA levels is not a critical predictor of GATA4 activity in model systems of hypertrophy. Over the course of experiments, mRNA levels of GATA4 remained stable in response to arginine‐8‐vasopressin infusion, nephrectomy in vivo and treatment with endothelin‐1 (ET‐1) in vitro,^29^, ^30^, ^31^ whereas exposure to phenylephrine (PE), isoproterenol or cardiomyocyte stretch in vitro were able to increase GATA4 mRNA levels.^32^, ^33^ On the other hand, treatment with cardiotoxic anthracyclines were associated with myocyte apoptosis and a reduction in both mRNA and protein levels of GATA4.^34^ Overall, these results indicate that activity of the myocardial GATA4 protein is preferably controlled by post‐transcriptional and post‐translational processes.^2^ The GATA family of proteins has shown high variance in their cellular protein stability and degradation rate. GATA2 protein has a relatively short half‐life (approximately 30 min) in comparison to GATA3 and GATA6, which have half‐lives over 3 hr when studied with cycloheximide, a protein synthesis inhibitor.^35^, ^36^, ^37^ However, the half‐life of GATA1 and GATA4 proteins far exceeds the other members of the GATA‐family, with half‐lives of greater than 6 hr.^36^, ^38^, ^39^ In general, the protein degradation rate plays an important role in protein displacement from the chromatin, especially in the case of related proteins, and therefore has a major impact on the establishment of transcription networks that control gene expression.^39^

The adult human heart has insufficient capacity to repair or regenerate cardiac cells after injury when a significant number of cardiomyocytes are lost. Scar formation and failure to regenerate the injured myocardium are the primary causes of the development of heart failure, arrhythmias and sudden death.^40^ Signaling pathways and regulatory mechanisms that are active during embryogenesis and are involved in heart growth and development may be used to repair the injured adult heart.^7^ The overexpression of cardiac GATA4 protein preserves cardiac function after cardiac injury by promoting increased angiogenesis and reduced fibrosis.^41^, ^42^, ^43^ Moreover, genetic enhancement of GATA4 protein was able to prevent cardiomyocyte apoptosis and drug‐induced cardiotoxicity.^44^ A study by Malek Mohammadi et al. demonstrated that high abundance of cardiac GATA4 by adenoviral gene transfer at postnatal days 1–7 markedly improved cardiac regeneration after cryoinjury and rescued the loss of regenerative capacity. Accordingly, larger myocardial scars were observed in cardiomyocyte‐specific GATA4 knockout mice after cryoinjury, accompanied by reduced cardiomyocyte proliferation and reduced myocardial angiogenesis.^45^, ^46^ In addition, molecular mechanisms of active cell populations responsible for the regenerative capacity of zebrafish have been linked to the expression of GATA4 within a week of cardiac injury. These results in zebrafish suggest the primary contribution and association of GATA4‐positive cells to heart regeneration and repair.^47^

### 2.1 GATA4 post‐translational modifications

The function of GATA4 protein is modified by enzymes through post‐translational processes, where one or more functional groups are covalently attached to or detached from the protein. Experimental studies show that post‐translational modifications of GATA4 involve the assignment of acetyl‐, phosphoryl‐, sumo‐, and ubiquitin moieties.^2^, ^48^, ^49^, ^50^ In cells, post‐translational modifications have an impact on several different functions of GATA4, involving nuclear localization, DNA binding affinity, coprotein association, and protein degradation.

The sequence of GATA4 protein conveys seven potential phosphorylation sites that are modified by enzymes, such as glycogen synthase kinase 3β (GSK‐3β), extracellular signal‐regulated kinases (ERK), and p38 mitogen‐activated protein kinase (MAPK), extensively reviewed by Suzuki and Zhou et al. In response to hypertrophic stimuli (e.g., ET‐1, PE, isoproterenol and myocyte stretch), activation of the MAPK kinase signalling cascade significantly augments GATA4 phosphorylation and DNA binding efficiency.^51^ The importance of phosphorylation was further evaluated by in vivo experiments with knock‐in mice carrying the homozygous GATA4‐S105A mutation, which demonstrated the compromised stress response of the myocardium.^52^ In addition, earlier studies have shown that GATA4 phosphorylation via the MAPK/ERK pathway at Ser105 gives the tendency to be more resistant to cellular degradation.^53^ In contrast, phosphorylation of the amino‐terminal part of GATA4 via activation of GSK‐3β resulted in increased export of GATA4 from the nucleus.^54^

Histone acetyltransferases such as p300 and cAMP‐response element binding protein (CREP) have been shown to induce the acetylation of specific lysine residues through physically interacting with GATA4.^50^, ^55^ Analogous to phosphorylation, GATA4 acetylation is similarly recognized as an imperative stimulus‐triggered mechanism that regulates cardiac hypertrophy by enhancing its DNA binding efficiency and transcriptional activity. Mutational analysis through alanine scanning by Takaya et al. identified four lysine residues (K311, K318, K320, K322) as targets of acetylation by p300. Mutation of all four residues blocked GATA4 acetylation and blunted cardiac hypertrophy induced by GATA4 overexpression, thus demonstrating the importance of GATA4 acetylation in the regulation of GATA4 transcriptional activity.^55^, ^56^ A recent study identified K311 (corresponding to K313 in the paper) as a primary target of acetyltransferases p300/CREP, with an enhanced cellular stability of acetylated GATA4.^57^ The study was carefully conducted to simulate the effect of loss‐of‐function by using lysine to arginine mutations for the optimal structural integrity of the mutated proteins. Other studies have also reported that acetylated GATA4 is more resistant to degradation, perhaps due to competition with lysine ubiquitination.^58^ Furthermore, a pharmacological study with trichostatin A demonstrated that acetylation of both GATA4 and histone residues are involved in the differentiation of embryonic stem cells into cardiac myocytes.^59^

GATA4 has been identified as a target protein for SUMOylation by small ubiquitin‐like modifier‐1 (SUMO‐1) and ubiquitination by the ubiquitin‐proteasome pathway.^38^, ^49^ Unlike the activation of the ubiquitin‐proteasome pathway leading to protein degradation, SUMOylation enhances GATA4 transcriptional activity through covalent binding of the SUMO motif exclusively to Lys366. In the cardiac context, the presence of protein inhibitor of activated signal transducer and activator of transcription‐1 (STAT‐1) and SUMO‐1 proteins triggered the enhanced SUMOylation of GATA4 and impacted both nuclear localization and cardiac gene activity. Active ubiquitination of GATA4 has been demonstrated in several physiological conditions, for example, hypoxia, hyperglycemia, and oxidative stress.^38^, ^60^, ^61^ Based on these observations, it appears that the ubiquitin‐proteasome pathway is the major degradation mechanism regulating the cellular turnover of GATA4 protein.

The study by Aries et al. demonstrated a specific case of the cellular effects of truncated GATA4 protein. Activation of caspase‐1 in cardiomyocytes by doxorubicin led to dominant‐negative GATA4 protein with a reduced ability to activate cardiac genes.^62^ Furthermore, it was shown that inhibition of caspase‐1 preserved transcriptional activity, reduced GATA4 protein degradation, and reduced myocyte cell death after doxorubicin exposure.

### 2.2 GATA4 chromatin occupancy

TFs regulate gene expression through coprotein assemblies together with basal transcriptional machinery by binding to specific *cis*‐regulatory sequences in gene promoters and enhancers. The tissue‐specific TF GATA4 prefers to bind to the DNA sequence (A/T)GATA(A/G) through its carboxy‐terminal zinc finger and is responsible for mediating site‐specific physical interaction with the DNA sequence. A number of essential cardiac‐expressed genes contain the binding sequence for GATA in their promoter, including atrial natriuretic peptide (ANP),^63^ B‐type natriuretic factor (brain natriuretic peptide [BNP]),^64^ α‐myosin heavy chain,^65^ β‐myosin heavy chain,^66^ cardiac troponin C,^67^ cardiac troponin I,^68^ and sodium‐calcium exchanger.^69^

In the adult heart, whole‐genome chromatin immunoprecipitation‐sequencing (ChIP‐seq) analysis with GATA4 antibody identified only 1,756 GATA4‐bound regions,^70^ whereas bioChIP‐seq in adult heart ventricles identified more than 15,000 binding sites for the high‐affinity FLAG‐biotin incorporated into GATA4,^71^ indicating a major difference in detection sensitivity related to the antibodies used in the experiment. During cardiac development, a high‐affinity ChIP‐seq system identified over 50,000 GATA4‐bound regions from the fetal heart ventricles. However, the less sensitive GATA4 antibody‐based chromatin immunoprecipitation ChIP‐seq identified 11,915 GATA4‐bound regions. Overall, the ChIP‐seq experiments indicate a dynamic change of GATA4 chromatin occupancy through normal heart development, in concert with its changing function. In the fetal heart, GATA4‐bound regions were predominantly located distal from the transcription start sites, while in the adult heart, a significant shift of GATA4 regions to proximal locations were observed. In the adult heart, pathological stress, such as chronic pressure overload, induced changes in GATA4 chromatin occupancy. The main stress‐induced differences of GATA4 recruitment were associated with completely new disease enhancers that were not occupied during development, as well as the partial reinitiation of the developmental program through GATA4 binding to a subset of fetal GATA4 enhancers.^71^

Chromatin remodeling controls gene expression by modifying the access of regulatory transcription machinery proteins to condensed genomic DNA. This genome‐wide remodeling process occurs via two different mechanisms, either by covalent histone modifications or by moving, ejecting, or restructuring the nucleosomes. Since gene activation is regulated in a multifaceted manner by the interplay of the TF network and the dynamic modifications of the chromatin landscape, as well as by the interference of microRNA (miRNAs), GATA4 chromatin occupancy alone was not directly associated to increased cardiac gene expression levels in fetal or adult heart. However, a number of studies have revealed a high correlation of genome‐wide enrichment of GATA4 binding regions, particularly to acetylated histone H3 at lysine 27 (H3K27ac), a major active transcriptional enhancer marker, together leading to a strong combined effect on gene activation.^17^, ^71^, ^72^, ^73^ Indeed, the binding strength of GATA4 did not correlate with the level of GATA4 target gene transcription assessed by ChIP‐seq, whereas the increased expression of GATA4‐bound genes was associated with higher H3K27ac enrichment at GATA4‐bound regions.

In human and mouse, there are approximately 2,000 TFs, more than 100 different modifications of histone residues, and approximately 700 miRNAs that modulate the mRNA profiles corresponding to approximately 20,000 genes. The TF complexes that are associated with GATA4 have a comparable dependency on cofactor binding and modulation by histone modifications, as well as on regulation by miRNAs. Therefore, tissue‐specific chromatin co‐occurrence with distinct subsets of TFs are preferred to allow a logical and systematic initiation/repression of transcription. Distinct cardiac TFs, such as NKX2‐5, TBX5, SRF, and MEF2A, in addition to enhancers such as p300, have been shown to localize together with GATA4 at chromatin regions and coregulate cardiac gene expression.^74^, ^75^ Even though these TFs are expressed in multiple tissues, ChIP‐seq experiments provide unbiased support for collaborative TF interactions in driving cardiac‐specific gene expression, which is especially linked to combinatorial localization and interactions between these cardiac TFs.

## 3 GATA4 ASSOCIATION TO THE CARDIAC TRANSCRIPTIONAL NETWORK

**Figure 2:**
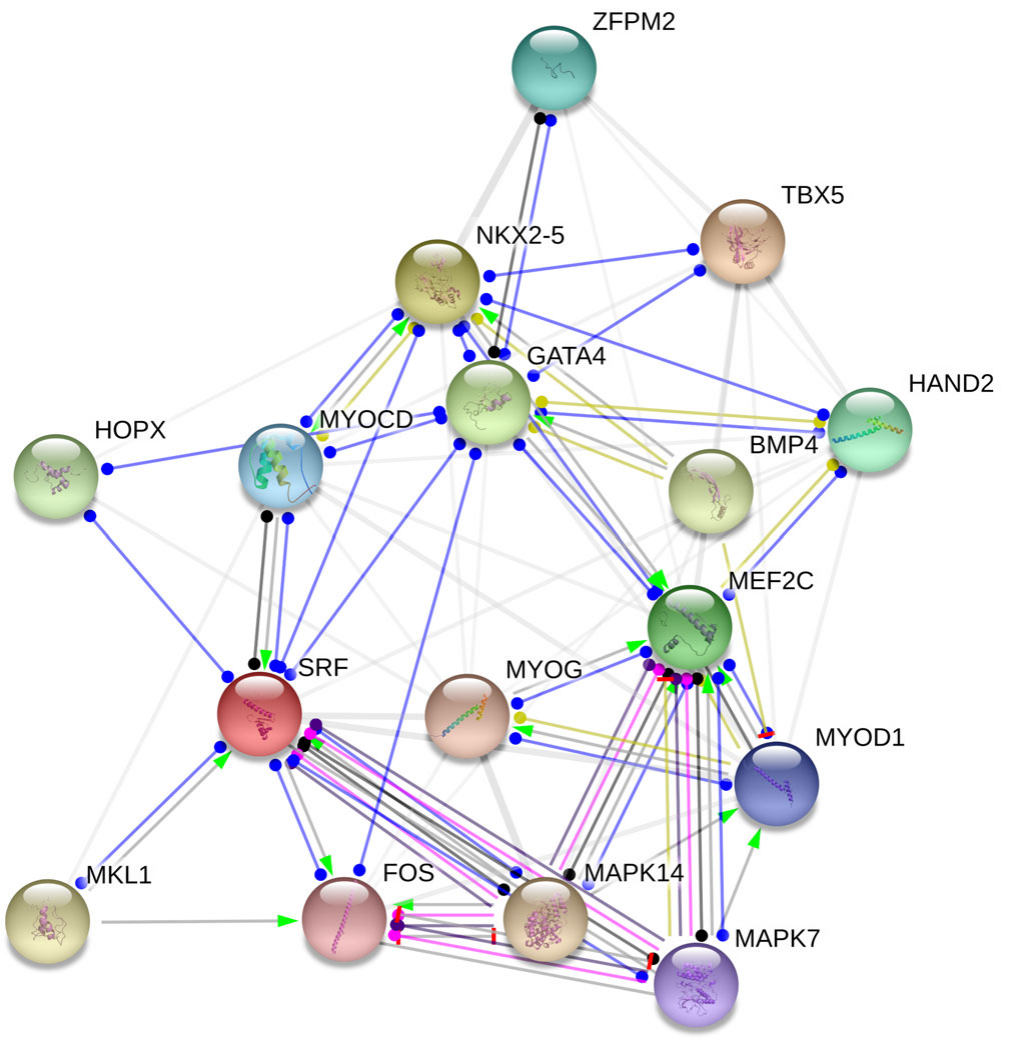
Cardiac protein association map derived from the STRING database illustrates the network of interactions for selected TFs; GATA4, NKX2‐5, MEF2C, HAND2, SRF, and TBX5. The associations are intended to be specific and meaningful, and thus, proteins jointly contribute to the shared functions. Interaction map color codes; blue indicates direct binding, purple indicates post‐translational modifications, yellow indicates transcriptional regulation, black indicates reaction, green arrow indicates activation and grey indicates the protein's indirect contribution to shared functions. Abbreviations: BMP4, bone morphogenetic protein 4 and HOPX, HOP homeodomain; FOS, FBJ murine osteosarcoma viral oncogene homolog; GATA4, GATA binding protein 4; HAND2, heart and neural crest derivatives expressed 2; MAPK7, mitogen‐activated protein kinase 7; MAPK14, mitogen‐activated protein kinase 14; MEF2C, myocyte enhancer factor 2C; MKL1, megakaryoblastic leukemia 1; MYOCD, myocardin; MYOD1, myogenic differentiation 1; MYOG, myogenin; NKX2‐5, NK2 homeobox 5; SRF, serum response factor; TBX5, T‐box 5; ZFPM2, zinc finger protein, multitype 2 (also known as FOG2)

Hereafter, we focus on GATA4 and its association with the interdependent cardiac TF system involving NKX2‐5, MEF2C, HAND2, SRF, and TBX5, which strictly controls the context‐dependent processes of cardiomyocyte development, maturation, and survival (Figure 2).^27^, ^45^ All these TFs regulate each other's expression and DNA binding preferences in a combinatorial manner, resulting in a buffering capacity of the network.^76^ Perturbation of the core TF network with chemical treatment or genetic alteration may lead to various cardiac phenotypes in mice, and mutations in humans have been associated with congenital heart defects. Although TFs are the main driving force for the precise control of gene expression, coregulators, epigenetic marks, and post‐transcriptional regulators, such as microRNAs, fine‐tune their expression and functional activity.^76^, ^77^ The combinatorial nature of TFs is facilitated by protein flexibility, which maximizes the specificity of promiscuous coprotein interactions. Due to combinatorial actions, a relatively small subset of TFs is able to control the transcriptional program of an entire cell.^78^, ^79^, ^80^

**Figure 3:**
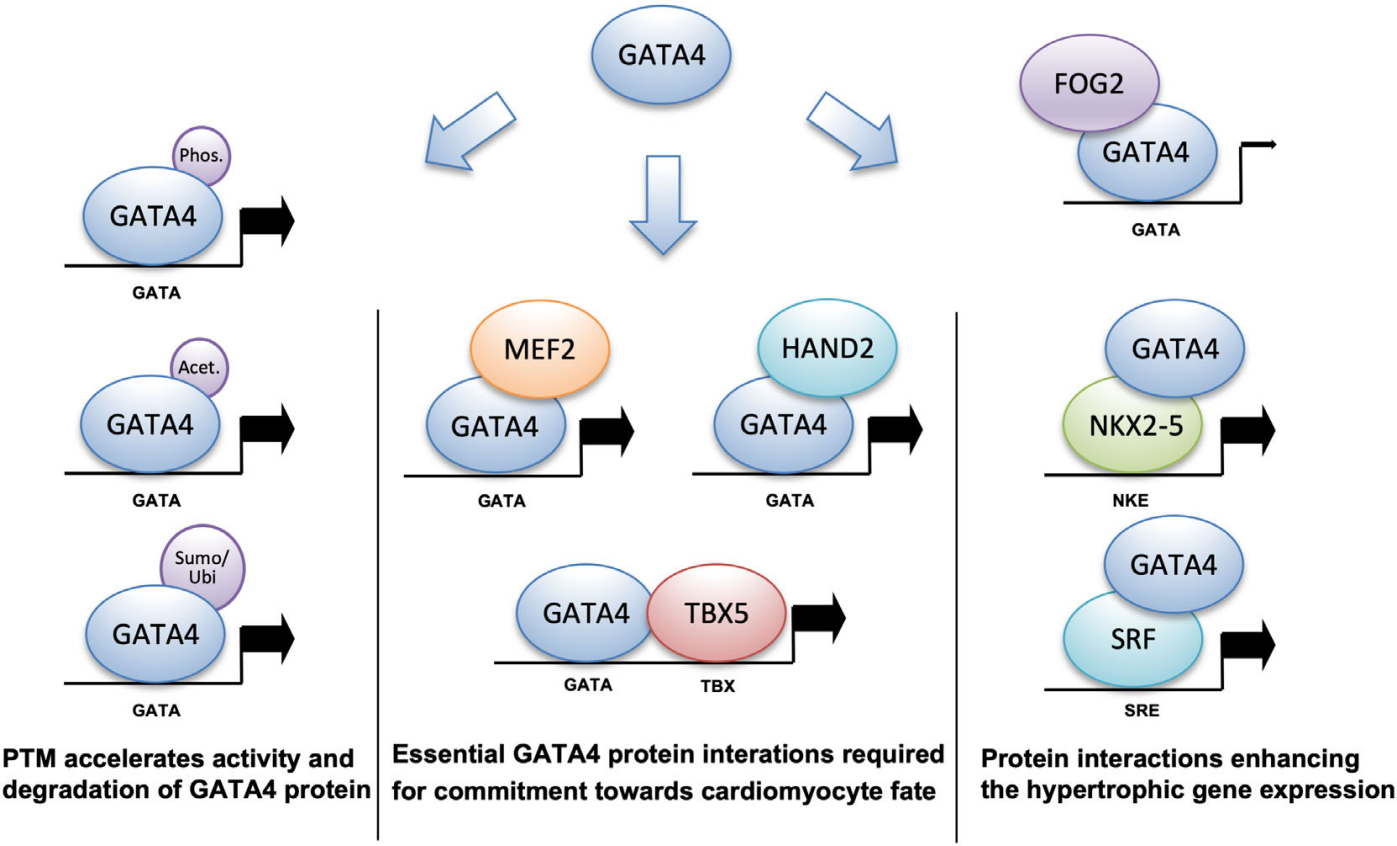
Cardiac transcriptional activity is regulated by interplay of the GATA4 transcription factor with several other TFs and post‐translational modifications. The vast majority of the protein associations of GATA4 are mediated by the C‐terminal zinc finger, while the N‐terminal zinc finger is responsible for interactions with the friend of GATA2 (FOG2). Cardiac specific heterotypic interactions and DNA occupation preferences for pair‐wise GATA4 ensembles are categorized based on experimental measurements of the protein and DNA binding modes. Specific context‐dependent GATA4 protein subconsortiums regulate both the commitment of stem cells toward the cardiac fate and hypertrophic gene expression in mature cardiac cells. Abbreviations: Acet., acetylation; GATA4, GATA binding protein 4; HAND2, heart and neural crest derivatives expressed 2; MEF2C, myocyte enhancer factor 2C; NKE, NK2 element; NKX2‐5, NK2 homeobox 5; Phos., phosphorylation; PTM, post‐translational modification; SRE, serum response element; SRF, serum response factor; Sumo/Ubi, sumoylation/ubiquitination; TBX5, T‐box 5

Heterotypic pair‐wise interactions of GATA4 have revealed that cofactors critical for direct cardiac reprogramming, such as MEF2C, HAND2, and TBX5, tend to synergistically activate the GATA cis‐regulatory element.^17^, ^18^, ^81^, ^82^ The dominant expression of either NKX2‐5 or SRF consequently lead to activation of the hypertrophic gene program, where synergy is driven through their corresponding DNA binding sites by the activation of a GATA4‐coprotein complex.^83^, ^84^ Diverse preprogrammed gene activation patterns are therefore consequences of operative selectivity arising from molecular conformations of the core factors at the promoter (Figure 3). Moreover, selected heterotypic GATA4 protein ensembles are capable of cooperating in cardiomyocytes through a single recognition DNA element, excluding the GATA4–TBX5 interaction, which requires binding elements for both TFs. Thus, understanding protein assembly and consequent gene regulation via an inside out approach, starting from pair‐wise heterotypic interactions as a core for more complex protein ensembles may further clarify the role of single TFs in gene regulation.

## 4 GATA4‐TARGETED SMALL MOLECULE INTERVENTIONS

There is currently only one report that presents research of direct GATA4‐targeted small molecule compounds. A study by El‐Hachem and Nemer utilized the integration of in silico and in vitro cell‐based screening assays to uncover charged small molecules that selectively and efficiently inhibited the DNA binding of GATA4.^85^ Two corresponding regions of the C‐terminal zinc finger domain of the NMR structure of chicken GATA1 and the crystal structure of murine GATA3 were utilized as highly conserved structural templates for the virtual screening campaign. A study by El‐Hachem and Nemer identified four compounds that inhibit GATA4 binding to DNA and in vitro blocked the activation of GATA4 downstream target genes and enhanced a mouse model of myoblast differentiation into myotubes. However, the use of negatively charged study compounds, which all contained zinc chelating moieties, were restricted entirely to in vitro assays due to the compounds' unoptimized and insufficient absorption, distribution, metabolism, and excretion (ADME) properties. The study was not able to confirm direct ligand binding to GATA4 or exclude possible ligand chelation to the zinc ion. Furthermore, since reduced protein and activity levels of GATA4 are linked to several adverse effects in cardiomyocyte differentiation, cardiomyocyte proliferation, cardiomyocyte apoptosis, and drug‐induced cardiotoxicity, application of DNA inhibitors of GATA4 protein may include potential risks for unfavorable cardiac effects in vivo.^44^, ^46^, ^52^, ^86^, ^87^, ^88^

### 4.1 The GATA4–NKX2‐5 interaction in cardiac development and hypertrophy

NK‐2 TF‐related, locus 5 (NKX2‐5 or Csx) is a cardiac specific homeobox gene family protein with a single helix‐turn‐helix motif responsible for binding to the specific consensus DNA sequence T(C/T)AAGTG. Evolutionarily conserved NKX2‐5 is a critical GATA4 cofactor and has an essential role in cardiac gene expression and normal heart development.^89^, ^90^, ^91^ To date, approximately 50 human mutations of NKX2‐5 have been identified that are associated with congenital heart defects responsible for the development of atrial septal defects, tetralogy of Fallot, and ventricular septal defects.^92^ Structural evaluations demonstrate that NKX2‐5 cooperates with cardiac TFs such as GATA4 and TBX5 through the homeodomain and its C‐terminal extension and synergistically promote cardiac gene expression, differentiation, and morphogenesis.^93^, ^94^, ^95^, ^96^ More specifically, mutational studies of NKX2‐5 have revealed the importance of residue Lys193 for the interaction with GATA4.^97^ Together GATA4 and NKX2‐5 directly interact and synergistically activate several cardiac genes including those encoding ANP and BNP.^2^, ^93^, ^98^ As the precise balance of the GATA4–NKX2‐5 interaction is important for cardiac gene expression and mechanical stretch‐induced cardiomyocyte hypertrophy,^32^ the functional modulation of their interactions could present a novel approach for cardiac repair in pathophysiological conditions.

Structural and functional data of key cardiac TF complexes are of major importance to enhance the understanding of molecular mechanisms and TF functions. Our previous study with GATA4–NKX2‐5 proteins showed that several single point mutations of GATA4 are capable of interfering or activating the GATA4–NKX2‐5 interaction,^99^ indicating that this PPI can be targeted by direct inhibition, activation or allosteric modulation. We established homology models of GATA4 and NKX2‐5 and evaluated the point mutations having critical effects on the GATA4–NKX2‐5 interaction. Preceding studies had shown that the second zinc finger and a C‐terminal extension were required for physical contact with NKX2‐5, and therefore, we mainly focused on these structurally stable regions.^93^, ^97^ Furthermore, the point mutations were selected to evenly cover the surface of the C‐terminal zinc finger of GATA4, excluding the amino acids needed for DNA binding. In total, 13 C‐terminal zinc finger point mutations (R264A, S269C, A271V, N272D, N272S, Q274H, S269C + Q274H, R283A, R283Q, E288G, E288K, M298Y, K299A) and four C‐terminal extension mutations (R319C, R319S, P321C, S327A) of GATA4 protein were generated together with two mutations produced in the N‐terminal zinc finger (V217Y, H234S). Since all mutations were located outside the antibody recognition site, western blots quantified the mutant protein levels produced in mammalian COS‐1 cells. The GATA4–NKX2‐5 interaction was assessed via coimmunoprecipitation with N‐terminal FLAG‐NKX2‐5 and analyzed by western blots using GATA4 and NKX2‐5 antibodies.^99^ Moreover, the DNA binding capacity of GATA4 mutations was evaluated. The results identify specific residues R264, N272, Q274, R283, M298, K299, and R319 within the C‐terminal zinc finger domain of GATA4 and its C‐terminal extension that were involved in physical and functional interaction with NKX2‐5. Integration of the experimental data with computational modeling suggests that the topology of the GATA4–NKX2‐5 interaction is reminiscent of that observed between the DNA binding domains of nuclear receptors. Nuclear receptors have two zinc fingers packed together with a conserved set of residues mediating stability of the domain. Our model predicts that the interaction between GATA4 and NKX2‐5 share a conserved architecture with the DNA binding domain of nuclear receptors.^99^ Another recent study employing molecular dynamics simulations suggests an alternative protein–protein interaction model for GATA4‐NKX2‐5 complex.^16^ Conversely, experimental studies do not find support for this theoretical observation.^93^, ^97^, ^99^

### 4.2 Compounds targeted to the GATA4–NKX2‐5 interaction

Based on the structural and functional data of the GATA4–NKX2‐5 interaction, an extensive chemical screening project was established incorporating computational and experimental biology to uncover compounds acting on key cardiac TFs. Targeting a protein–protein interaction with a small molecule is challenging due to the large surface area involved in protein–protein binding and the lack of clear binding pockets at many protein–protein interfaces. Therefore, the discovery approach consisted of several recurring methods, including GATA4‐NKX2‐5 co‐immunoprecipitation and luciferase reporter assays specifically developed to explore chemical agents possessing either agonistic or antagonistic effects on GATA4–NKX2‐5 transcriptional synergy.^100^ Fragment‐based screening, virtual screening docking and pharmacophore methods, were accordingly employed to identify primary hit compounds. Initial low molecular weight fragments were extensively modified with different structural options through chemical synthesis to improve the affinity of the molecular scaffold. The screening project resulted in synergy inhibitory compounds, including *N*‐[4‐(diethylamino)phenyl]‐5‐methyl‐3‐phenylisoxazole‐4‐carboxamide (IC_50_ 3 μM), and synergy activator compound *N*‐(4‐chlorophenyl)‐5‐methyl‐*N*‐(4‐methyl‐4,5‐dihydrothiazol‐2‐yl)‐3‐phenylisoxazole‐4‐carboxamide. The structure–activity relationship of inhibitory compounds demonstrated that ligand affinity associates to the two heavy atom linkers and hydrogen acceptors next to the five‐member ring, while the agonistic effect was achieved by introducing a substituted five‐member ring into the amide bond. The most potent inhibitory and activator compounds were tested in various hypertrophy assays. In cardiomyocyte cultures, the compounds either augmented or inhibited ET‐1‐ and PE‐induced increases in ANP and BNP gene expression, in line with inhibition or activation of the GATA4–NKX2‐5 interaction.^100^, ^101^ Moreover, the inhibitory compound 3i‐1000 significantly reduced mechanical stretch induced hypertrophic growth reflected by an increase in cardiomyocyte cell size and ANP and BNP mRNA levels in response to mechanical stretch.

In vivo experiments showed that inhibition of GATA4–NKX2‐5 transcriptional synergy has beneficial effects on cardiac function and gene expression in several experimental models of myocardial ischemia and pressure overload.^101^, ^102^ Echocardiographic evaluation showed significant improvement in left ventricular ejection fraction and fractional shortening and significant attenuation of myocardial structural changes in 3i‐1000 treated mice after myocardial infarction. Accordingly, the increase of natriuretic peptide gene expression caused by myocardial infarction and the increase in ANP gene expression induced by myocardial ischemia reperfusion injury were significantly decreased by 3i‐1000 in mice. Furthermore, compound 3i‐1000 improved cardiac function in an experimental model of angiotensin II‐mediated hypertension in rats. Optimization of primary modulators of GATA4–NKX2‐5 interactions are ongoing to improve metabolite and safety profiles and determine the direct protein targets of transcriptional synergy inhibition associated with molecular mechanism(s) of action.^103^

It is also noteworthy to point out other challenges that would come from attempting to disrupt GATA4 activity. Beyond the fact that GATA4 is an essential TF in cardiac cells, it is also expressed and functional in many other tissues, prominent among these the pancreas, liver, and lung. In addition, the targeting should be specific for GATA4 and not interfere with activities of sister genes, particularly GATA5 and GATA6. Therefore, strategies to limit off‐targeting of other organs and other TFs should be developed. One opportunity to limit off‐targeting of other organs would be to develop nanotherapies targeted to the injured region of the myocardium with the potential for spatial and temporal control of drug delivery.^102^ Overall, to consider GATA4 a bonafide drug target, the challenges of selectively targeting GATA4 and the injured myocardium should be addressed in more detail.

## 5 CONCLUSION

Current therapies for the treatment of myocardial remodeling are shown to attenuate symptoms and prolong lifespan by reducing the workload of the heart, for example, angiotensin converting enzyme inhibitors, angiotensin receptor blockers, beta‐blockers, and mineralocorticoid receptor antagonists. However, prognosis of patients remains poor with present‐day pharmacological treatments. Cell transplantation and virus‐mediated TF delivery therapies aim to replace damaged cells with new functional cardiomyocytes by utilizing hiPSC‐derived cardiomyocytes and stem cell transplantation, and by reprogramming nonmuscle cells towards cardiomyocytes. However, providing therapeutic value with cell transplants and virus‐carried TFs remains a challenge due to poor integration of new cells into the heart and the safety concerns of gene therapy. Modulation of cardiac signaling pathways and TF networks by chemical entities may represent another viable option for therapeutic intervention. Nonimmunogenic and cost‐effective small molecules possess significant advantages of cell permeability and management of standardized industrial production and quality assurance.

Our recent results (3xNKE reporter assay, ET‐1 and PE‐stimulated gene expression of ANP and BNP, hypertrophic cell surface growth in cardiomyocytes, and immunoprecipitation assays) demonstrate that compounds acting on GATA4‐NKX2‐5 transcriptional synergy can modulate the hypertrophic response in cardiomyocytes in vitro. In addition, we have shown that 3i‐1000, a small molecule inhibitor of GATA4‐NKX2‐5 transcriptional synergy, provides cardioprotective effects in vivo, indicating that modulators of protein–protein interactions of key TFs may present novel pharmaceuticals for cardiac remodeling and repair.

## CONFLICT OF INTERESTS

M.J.V. and H.J.R. are inventors in a pending patent application “Pharmaceutical compounds” (PCT/FI2017/050661).
